# Progress in multiaxial coupling mechanisms of osteoporosis: insights from systems medicine on immune-inflammatory interactions, hormonal regulation, and metabolic imbalance

**DOI:** 10.3389/fendo.2025.1705040

**Published:** 2026-01-05

**Authors:** Manli Yan, Miyang Yang, Yaoqian Cai, Baoqing Zhang, Dingkun Lin, Xiang Li

**Affiliations:** 1 Second Clinical Medical College, Guangzhou University of Chinese Medicine, Guangzhou, China; 2 Sun Yat-Sen University, Zhongshan School of Medicine, Guangzhou, China; 3 Department of Orthopedics, Guangdong Provincial Hospital of Chinese Medicine, Guangzhou, China; 4 Orthopaedic Specialty Hospital, Guangdong Provincial Hospital of Chinese Medicine, Guangzhou, China; 5 Department of Orthopaedic Teaching and Research, Guangdong Provincial Hospital of Chinese Medicine, Guangzhou, China

**Keywords:** bone–immune–endocrine–metabolic axis, hormonal regulation, metabolic disorder, osteoimmunology, osteoporosis

## Abstract

Osteoporosis (OP) is increasingly recognized as a systemic disorder involving crosstalk among immune-inflammatory, endocrine, and metabolic axes, rather than a condition driven solely by bone-mineral imbalance. This review synthesizes recent advances in understanding these axes and their coupling mechanisms. The immune-inflammatory axis, centered on Th17/Treg imbalance and cytokines such as IL-17 and IL-35, regulates osteoclastogenesis and osteogenesis while linking autoimmune diseases to bone loss. The endocrine axis, encompassing sex hormones, thyroid/adrenal/parathyroid hormones, and receptors such as LGR4, modulates bone remodeling through complex signaling networks and circadian rhythms. The metabolic axis, particularly glucose-lipid-uric acid homeostasis, influences bone fragility via energy metabolism, oxidative stress, and gut microbiota interactions. We propose that OP is best understood as a network disorder manifesting in the skeleton, necessitating a shift from single-target to multi-axis systems medicine approaches. However, much of the mechanistic evidence derives from preclinical models and observational studies; prospective clinical validation remains essential. Future research should prioritize elucidating inter-axis communication in human cohorts and developing integrated, biomarker-stratified therapeutic strategies to advance precision management of osteoporosis.

## 1 Introduction

**Figure 1:**
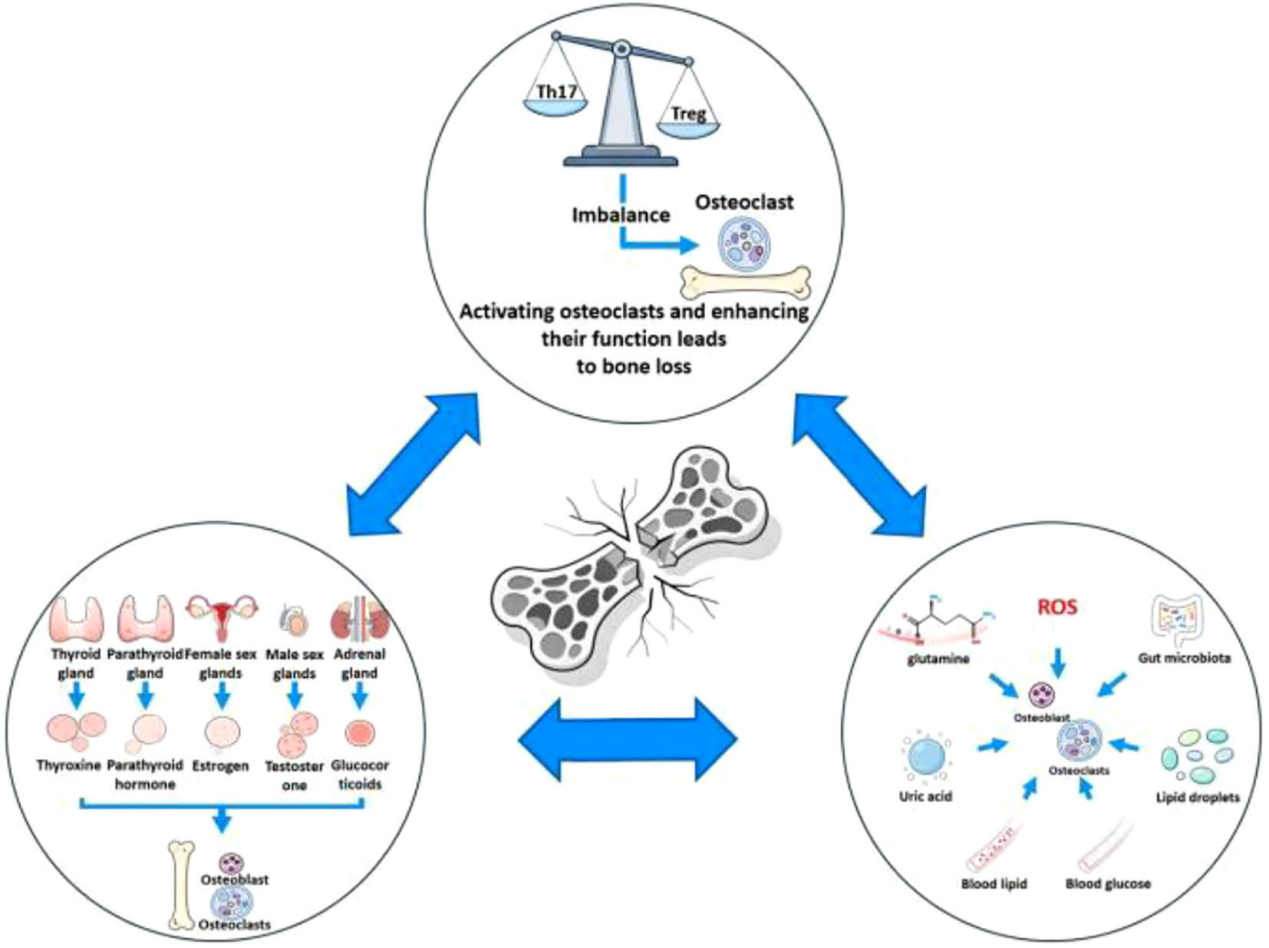
Multi-axial regulatory framework of osteoporosis.

Osteoporosis (OP) is a prevalent bone disorder worldwide, characterized by a reduction in bone mass and deterioration in bone properties, which collectively heighten bone fragility and the risk of fracture. In 2019, approximately 8.14 million women and 6.11 million men aged 50 years and older experienced hip fractures globally (1), imposing substantial social and economic burdens. Traditionally, the pathophysiological model of OP has centered on bone-mineral metabolism. However, advances in systems biology have broadened our perspective, leading to the recognition of a multi-axial framework involving bone, immune, endocrine, and metabolic axes (**Figure 1**). Interactions among immune-inflammatory responses, hormonal regulation, and metabolic pathways are now understood to play pivotal roles in bone resorption, turnover, and homeostatic remodeling. This review synthesizes recent progress in understanding these three axes, discusses their translational implications, and aims to guide future research and clinical practice in OP prevention and treatment. It is noteworthy that much of the evidence supporting the multi-axis framework discussed in this paper, particularly the mechanistic details, primarily comes from experimental models and observational studies. In contrast, prospective clinical intervention data based on human subjects are relatively scarce. This suggests that when interpreting and applying these findings clinically, it is crucial to consider the limitations of the available evidence.

## 2 Immuno-inflammatory axis: osteoimmunological mechanisms and systemic modulation

### 2.1 Th17/Treg cell balance: a central node linking inflammatory factor networks and bone metabolic coupling

Osteoimmunological studies indicate that excessive bone destruction is predominantly driven by increased osteoclast-mediated resorption, which is tightly regulated by CD4+ T cells infiltrating skeletal lesion sites (2). Among the CD4+ T cell subsets, Th17 and Treg cells exert pivotal effects on the maintenance of bone metabolic equilibrium (3). Substantial evidence has demonstrated that (4) Th17/Treg imbalance is closely associated with heightened osteoclast differentiation, constituting an immunological foundation for inflammation-driven bone loss.

Th17 cells modulate the RANKL/RANK/OPG signaling axis both directly and indirectly, while also promoting the secretion of inflammatory cytokines such as TNF-α, IL-1, IL-6, and IL-17 (4, 5). IL-17 (3, 5) promotes the differentiation of mesenchymal stem cells into osteoblasts and induces the osteogenic differentiation of osteoprogenitor cells. On the other hand, it stimulates osteoblasts to secrete RANKL, thereby enhancing bone resorption, and upregulates RANK expression on osteoclast precursors, increasing their sensitivity to RANKL. Several studies suggest that (4) IL-17 plays a pivotal role in bone destruction in murine models of rheumatoid arthritis. It is noteworthy that the regulatory role of IL-17 in bone metabolism remains subject to debate across different studies. Nevertheless, available evidence suggests that (4, 6) anti-IL-17 interventions can effectively mitigate bone degradation induced by estrogen deficiency, highlighting IL-17 signaling as a potential target for OP prevention and therapy.

Moreover, IL-17 (7) plays a critical role in regulating energy metabolism during osteoclast differentiation, with its pro-osteoclastogenic effects being highly dependent on glutamine availability. IL-17 enhances energy production to promote osteoclast formation; however, when glutamine is depleted or its cellular transport is pharmacologically inhibited, this effect is markedly attenuated. *In vivo* studies (7) have further validated this dependency: in ovariectomized (OVX) animal models, administration of IL-17 exacerbated bone loss, while treatment with the glutamine uptake inhibitor V9302 partially mitigated this process. Supplementing α-ketoglutarate, a downstream product of glutamine metabolism, partially reversed the protective effect of V9302, indicating that the bone-resorptive action of IL-17 is mediated through the glutamine metabolic pathway. A deeper investigation revealed that (8) the development of OP in OVX rat models is associated with alterations in the gut microbiota, particularly a reduction in lactobacilli, which is closely linked to the onset of OP. Additionally, the serum levels of TNF-α, IL-6, and IL-17 were found to be elevated.

Conversely, Treg cells (4) exert a protective influence on bone by downregulating RANKL and M-CSF levels, and secreting immunosuppressive cytokines such as IL-10, TGF-β, and IL-35, thereby effectively inhibiting osteoclastogenesis. As a key product of Treg cells, IL-35 (3) not only reduces IL-17 release—suppressing Th17-driven osteoclast differentiation—but also promotes mesenchymal stem cell proliferation, inhibits apoptosis, and significantly enhances osteogenesis while concurrently suppressing mesenchymal stem cell adipogenic differentiation. Thus, IL-35 contributes to dynamic regulation of bone mass.

Emerging evidence has revealed that IL-35 (9) activates the Wnt/β-catenin signaling pathway, increasing the expression of osteogenic markers such as β-catenin and Axin2, while downregulating adipogenic markers PPAR-γ and C/EBPα. This suggests that IL-35 (10) may play a critical role in orchestrating the balance between osteogenic and adipogenic differentiation of progenitor cells via the Wnt/β-catenin-PPARγ axis. IL-35 treatment (9) not only robustly promotes osteogenesis, but also effectively suppresses adipogenesis, highlighting its potential therapeutic utility in both OP and obesity.

In summary, Th17 and Treg cells synergistically regulate bone resorption and formation by modulating inflammatory cytokines, bone-associated signaling pathways, and stem cell differentiation networks, thereby facilitating a tight coupling between immune function and bone metabolism. From a systems biology perspective, both IL-17 and IL-35 act not only on bone cell metabolism, energy regulation, and differentiation equilibrium, but also exemplify the multi-axial interactions among bone, immune, endocrine, and metabolic pathways. Immunological imbalance, particularly the persistent imbalance between Th17 and Treg cells, primarily exacerbates bone loss by inducing a chronic inflammatory microenvironment, promoting osteoclastogenesis, and inhibiting osteoblast function. Existing evidence suggests that (11–14) immune imbalance alone may not be sufficient to independently induce clinically significant OP. Instead, it acts as a key pathological amplifier, working synergistically within a multi-axis network (including metabolic and endocrine disturbances) to drive bone homeostasis dysregulation. This mechanism is especially prominent in the context of autoimmune diseases and aging, forming a pathological network of multifactorial interactions in OP.

### 2.2 Multiscale mechanisms linking autoimmunity and osteoporosis

Epidemiological studies have suggested a significant association between various autoimmune disorders and OP (15), although the underlying mechanisms remain incompletely defined. On one hand, chronic immune dysregulation in autoimmune conditions specifically impairs bone remodeling and causes bone loss (16); on the other, autoimmune diseases are often accompanied by disturbances in nutrient metabolism and absorption (15, 17), which further jeopardize skeletal homeostasis and contribute to OP development. Additionally, pharmacologic agents commonly prescribed in the management of autoimmune diseases, such as glucocorticoids, may exacerbate bone loss (18, 19).

Building on these observations, recent Mendelian randomization analyses utilizing European and East Asian GWAS datasets have confirmed (20) a causal relationship between inflammatory bowel disease, decreased bone mineral density, and increased risk of OP, uncovering disease subtype-specific effects on site-specific bone metabolic outcomes. These genetic findings provide causal evidence for the associations observed in epidemiological studies and guide us to further explore the downstream cellular and molecular mechanisms. Future studies with larger sample sizes and higher precision are expected to further elucidate the mechanisms linking other autoimmune diseases to bone metabolism abnormalities.

It is noteworthy that the interaction between immunity and bone is particularly pronounced in the elderly population, with common upstream driving factors likely stemming from immune aging (21, 22). In this context, rheumatoid arthritis (especially late-onset rheumatoid arthritis) and age-related OP frequently coexist in older individuals. The mechanisms involved include (22): a decline in sex hormone levels and the immune regulatory factor IL-2, an increase in serum autoantibodies and pro-inflammatory mediators (such as TNF-α and IL-6), upregulation of key bone metabolism regulators such as RANKL, DKK1, and sclerostin, and imbalance in the IL-33/IL-31 axis. The chronic low-grade inflammatory state associated with immune aging shapes a specific inflammatory-endocrine microenvironment, thereby forming the core pathological basis linking aging, autoimmunity, and bone loss.

At the cellular level, notable progress has been made in leveraging extracellular vesicles, especially exosomes, derived from mesenchymal stem cells as promising candidates for cell-free therapeutic applications. As nano-scale biological vectors, exosomes (23) mediate intercellular communication by transferring genetic and metabolic information, and they modulate immune responses and inflammation, playing critical roles in the regulation of innate immunity. Accumulating evidence demonstrates that (24) exosomes display significant therapeutic potential in autoimmune and rheumatic diseases, including OP. Specifically, exosomes contain osteogenic signaling molecules, such as components of the eukaryotic initiation factor-2 pathway (23), which promote bone formation. Additionally, exosomes can protect cells from apoptosis under stressors such as hypoxia, thereby preserving bone tissue homeostasis. Therefore, the exosomal mechanism has emerged as a specific pathway linking immune dysregulation with bone metabolism abnormalities.

Beyond extracellular vesicle-mediated communication, direct molecular targeting intervention studies have also revealed new mechanisms. Molecularly targeted pharmacological interventions have opened new avenues for managing bone metabolic disorders. Inhibitors of CBP/p300 have recently emerged as significant epigenetic targets in the context of inflammation and autoimmune pathologies. Notably, the CBP/p300 inhibitor A-485 (25) has been shown to effectively suppress osteoclast differentiation, thereby preventing osteoporotic bone loss and expanding the therapeutic repertoire for skeletal metabolic diseases. In *in vitro* experiments (26), A-485 promotes osteogenic differentiation and improves mitochondrial function by activating GLUD1 deacetylation.

Taken together, both large-scale epidemiological analyses and mechanistic studies at the cellular and molecular levels reveal the complexity and heterogeneity of autoimmune-associated disruptions in bone metabolism. Precision intervention targeting multiple signaling axes holds promise for providing a more robust scientific foundation and innovative strategies for the prevention and treatment of OP (**Tables 1**, **2**).

**Table 1:** Potential intervention strategies targeting the immune-inflammatory axis and their molecular mechanisms.

Intervention type	Intervention strategy	Core target/pathway
Biological agent	Anti-IL-17 therapy	IL-17 drives osteoclast differentiation by enhancing glutamine-dependent energy metabolism; anti-IL-17 therapy blocks this signaling to alleviate estrogen deficiency-induced bone degradation
Biological agent	IL-35 administration	Activates Wnt/β-catenin pathway and inhibits PPARγto regulate osteogenic/adipogenic differentiation balance
Cell therapy	MSC-derived exosomes	Mediates intercellular communication (containing eIF2 pathway components) to regulate immunity and promote osteogenesis
Small molecule inhibitor	CBP/p300 inhibitor (e.g., A-485)	Suppresses p300/CBP activity to inhibit osteoclast differentiation

**Table 2:** Potential intervention strategies targeting the hormonal regulatory axis and their molecular mechanisms.

Hormonal regulatory sub-axis	Intervention strategy	Core target/pathway
Sex hormone axis	Estrogen (e.g., 17β-estradiol)	Activates ESR1-Keap1-Nrf2 axis, alleviates oxidative stress, promotes osteoblast survival and differentiation, and maintains bone homeostasis
Adrenal axis	Columbianadin	Regulates RANK/RANKL/OPG signaling balance, inhibits excessive osteoclast activation, and improves glucocorticoid-induced osteoporosis
Adrenal axis	Selenium-enriched egg white protein	Alleviates adverse effects of glucocorticoids on bone metabolism and reduces disease phenotypes through gut microbiota-mediated Htr1b inhibition pathway
Adrenal axis	SIRT3 targeting	Regulates mitophagy-dependent ferroptosis, protects osteoblast function, and prevents glucocorticoid-induced osteoporosis
Parathyroid hormone axis	Novel PTH analogs (e.g., ^R25C^PTH(1-34))	Activates PTH1R, antagonizes Hippo pathway (via Src-YAP), promotes osteoblast differentiation, and reduces the risk of calcium-phosphate metabolic abnormalities
Parathyroid hormone axis	combined with β-blockers (e.g., propranolol)	Attenuates sympathetic nervous system’s negative regulation on bone metabolism, enhances PTH’s osteogenic effect, and promotes osteoporotic fracture healing and systemic bone mass maintenance
Parathyroid hormone axis	3D-printed macroporous titanium alloy scaffold + PTH-related peptide	Constructs bone regeneration microenvironment, enhances osseointegration efficiency between implant and bone tissue under osteoporotic conditions, and promotes local bone repair

## 3 Endocrine regulation axis: advances in endocrine-bone coupling and chronobiology

### 3.1 Gonadal hormone axis: sex dimorphism, reproductive events, and skeletal homeostasis

A growing body of evidence demonstrates pronounced sexual dimorphism in OP (27). Postmenopausal women experience accelerated bone loss due to a sharp decline in estrogen levels, resulting in higher disease prevalence compared to men. In contrast, men typically exhibit greater bone strength and reduced risk for primary OP, yet are more susceptible to secondary forms (27), which are associated with higher rates of disability and mortality following fractures.

Mechanistic studies have revealed that (28, 29) estrogen deficiency primarily impairs the oxidative stress resilience of bone marrow stromal/stem cells by downregulating Nrf2 expression, leading to heightened oxidative stress, reduced cell survival, and diminished osteogenic differentiation and bone formation. Recent research indicates that 17β-estradiol (28) exerts its protective effect via the ESR1–Keap1–Nrf2 axis: ESR1 competes with Nrf2 for Keap1 binding, thereby inhibiting Nrf2 degradation, activating the antioxidant response, and upregulating transcription of Tmem119. Correspondingly, Messeha et al. propose that (30) Nrf2 activation is a promising therapeutic target for age-related OP and highlight the potential of various natural compounds (such as flavonoids) as safe alternatives with both antioxidant and anti-osteoporotic properties. Furthermore, as a member of the nuclear receptor superfamily, estrogen receptor α (ESR1) (31) is not only involved in the reproductive system but is also widely expressed in immune cells, bone tissue, and metabolic organs, where it plays a crucial role in regulating immune responses and maintaining metabolic homeostasis. Estrogen signaling is cell-type and microenvironment-dependent, and can differentially regulate cellular bioenergetics, inflammation, and immune responses. Estrogen-related endocrine disruptors can also modulate macrophage activity by altering cellular bioenergetics.

Compared to women, men typically do not experience significant gonadal dysfunction in the short term as they age. Studies have shown that (32) transgender men receiving testosterone therapy do not experience bone mass loss, even under ovarian estrogen suppression, underscoring the critical role of testosterone in preserving male bone mass. Data also suggest that (33) the association between serum testosterone levels and bone loss is more pronounced in older men, while no such correlation is observed in younger men. Testosterone replacement therapy has also been shown to increase bone mineral density, although its effect on fracture risk remains inconclusive. Testosterone-androgen receptor signaling (34) can delay bone degradation in elderly men by upregulating osteoblast extracellular tenascin-C.

Furthermore, recent studies have yielded nuanced insights regarding the gonadal axis and skeletal homeostasis. In a cross-sectional analysis of individuals with type 2 diabetes mellitus, Li et al. (35) observed that serum levels of dehydroepiandrosterone and its sulfate—rather than testosterone or estradiol—were positively correlated with higher bone mineral density and reduced OP risk in postmenopausal women, an association not observed in men over 50. These findings await confirmation in larger and more diverse cohorts. In addition, Koh et al. (36) provided evidence that follicle-stimulating hormone directly influences bone metabolism independently of sex steroid levels, suggesting multifaceted roles in aging and frailty.

Pregnancy- and lactation-associated osteoporosis (PLO) has also attracted increasing attention (37), with most cases occurring within three months postpartum (38, 39). High bone turnover during pregnancy and lactation accommodates the calcium demands of the fetus and infant (37). While physiological upregulation of intestinal calcium absorption during gestation helps preserve maternal bone mass, insufficient dietary calcium intake leads to increased bone resorption and elevated PLO risk (37). The rapidly shifting endocrine milieu during pregnancy and lactation further perturbs bone homeostasis (40), constituting another important etiological factor. Currently, no standardized clinical guidelines exist for the management of PLO (38); given the unique characteristics of affected individuals, personalized and multifactorial treatment approaches are required. High-quality studies are urgently needed to establish evidence-based interventions and identify novel therapeutic avenues for patients with PLO.

### 3.2 Thyroid axis: functional abnormalities, hormone sensitivity, and skeletal metabolic risk

Thyroid hormones (41) exert their physiological functions in bone primarily via thyroid hormone receptor α expressed in osteoblasts, and further stimulate osteoclast-mediated bone resorption through osteoblast-driven cytokine signaling pathways. These actions are crucial for maintaining adult skeletal architecture and strength, which underlines the tight relationship between thyroid status and bone health outcomes.

Overt hyperthyroidism (42) is well recognized as a cause of increased bone turnover and accelerated bone loss; in this state, bone resorption exceeds bone formation, resulting in decreased bone mineral density (BMD), heightened risk of OP, and increased susceptibility to fractures. Subclinical hyperthyroidism—whether endogenous or due to TSH suppression (including overtreatment with levothyroxine)—also poses a detrimental impact on bone integrity (41). By contrast, the association between hypothyroidism and bone homeostasis remains controversial. Some evidence suggests that (43) TSH may exert a direct protective effect on bone independently of thyroid hormones themselves. Recent Mendelian randomization studies in European populations indicate that (42) thyroid-related diseases may causally increase the risk of OP. Specifically, TSH-mediated hypothyroidism and FT4-mediated hyperthyroidism both show causal links with OP; moreover, genetically elevated serum TSH levels are associated with a lower risk of fractures in men (44), implying a direct bone-protective role for TSH, although its independence requires further mechanistic clarification.

The concept of “thyroid hormone sensitivity”—often quantified by indices such as TSHI, TT4RI, TFQI, and FT3/FT4 ratios—has also attracted increasing attention. A cross-sectional study by Liu et al. found that (45), among older adults with normal thyroid function, impaired thyroid hormone sensitivity was independently associated with OP and fractures. This suggests that, in addition to absolute levels of TSH and FT4, the phenotype of hormone sensitivity could provide incremental value for skeletal risk assessment. However, given that the study was cross-sectional, causal relationships must be further verified in longitudinal cohorts and causal inference models.

### 3.3 Adrenal axis: glucocorticoid-mediated bone metabolic imbalance and emerging interventional approaches

Dysregulation of the hypothalamic–pituitary–adrenal (HPA) axis is a significant, yet often underrecognized, modifiable risk factor for skeletal health. Prolonged use of exogenous glucocorticoids, endogenous hypercortisolism, and primary aldosteronism are all endocrine states associated with increased bone fragility and a substantial burden of fractures. Animal studies have indicated that (46) the implantation of slow-release corticosterone (CORT) pellets in female C57Bl/6J mice, designed to attenuate the rhythmic fluctuations of CORT levels without inducing Cushing’s syndrome, results in a flattening of the CORT rhythm. This flattening leads to a reduction in the volume and thickness of both cortical and trabecular bone. Mice injected with supraphysiological doses of CORT were still able to maintain bone structure during endogenous glucocorticoid peaks. These findings suggest that the loss of the physiological glucocorticoid secretion rhythm alone can induce an osteoporotic phenotype. Moreover, disruption of the circadian rhythm also affects the secretion of hormones such as growth hormone and testosterone (47), metabolic factors such as insulin sensitivity (48), and interferes with inflammatory responses and phagocytosis pathways in macrophages (49), thereby influencing bone mass from multiple angles.

Mechanistically, glucocorticoids adversely affect bone remodeling through multiple pathways (50, 51). Direct actions on osteoblasts, osteoclasts, and osteocytes suppress bone formation while promoting bone resorption. Indirectly, they inhibit the growth hormone and gonadal axes, decrease intestinal calcium absorption, and contribute to glucocorticoid-related myopathy, all of which exacerbate bone loss. Additionally, glucocorticoids negatively influence the parathyroid hormone–vitamin D axis (51), a factor of potential clinical significance.

Epidemiological data demonstrate that both systemic and inhaled glucocorticoids, especially with prolonged use or higher cumulative doses, are linked to increased risk of OP and fragility fractures (52). Observational studies further suggest that prolonged, high-potency topical glucocorticoid therapy, particularly when used over large skin areas or under occlusion, can also elevate the incidence of OP and major fractures (52, 53).

Robust evidence supports the use of bisphosphonates in reducing the risk of vertebral fractures (54); in pediatric cases of glucocorticoid-induced OP, these agents have also been found to improve bone mineral density and relevant Z-scores (55). Nevertheless, bisphosphonate therapy carries certain adverse effect risks, necessitating a careful balance between efficacy and safety in clinical decision-making.

Building on these insights, novel therapeutic approaches are under investigation. Animal experiments have shown that Columbianadin (56) alleviates glucocorticoid-induced OP by modulating the RANK/RANKL/OPG signaling axis. Targeting SIRT3 (57) to regulate mitochondrial autophagy–dependent ferroptosis may present another avenue for the prevention of glucocorticoid-induced OP. Buttgereit and colleagues (58) are exploring the development of selective glucocorticoid receptor modulators to optimize therapeutic efficacy and safety, though further research is needed to validate these strategies.

### 3.4 Parathyroid hormone: central regulator of bone anabolism and advances in engineering-based optimization

Parathyroid hormone (PTH) (59) regulates serum calcium and inorganic phosphate homeostasis through coordinated actions on bone and kidneys. In skeletal tissue, PTH directly influences osteoblasts and osteocytes, orchestrating bone remodeling, and also governs the fate of bone marrow stromal cells by directing their differentiation toward either the osteogenic or adipogenic lineage. Recent studies have revealed that, on one hand, PTH (59) antagonizes the Hippo signaling pathway through Src-dependent YAP stabilization, thereby enhancing the osteogenic differentiation potential of bone marrow stromal cells. On the other hand, PTH (60)induced transcriptional activation of RANKL is modulated by SIK2/3 and mediated via CRTC2/3, with this effect reliant on the activity of protein phosphatases 1, 2, 4, and 5.

Current strategies for modulating PTH anabolic pathways focus on system regulation, ligand engineering, and tissue engineering. At the systemic level, non-selective β-blockers such as propranolol (61, 62) attenuate sympathetic signaling, thereby diminishing its antagonistic effect on PTH’s anabolic actions and enhancing PTH-mediated bone formation after osteoporotic fractures. Studies have shown that co-administration of PTH with propranolol (61) markedly promotes systemic bone formation and accelerates fracture healing in ovariectomized mice. In terms of ligand engineering, novel PTH peptides (63, 64)can activate PTH1R both *in vitro* and *in vivo*, stimulating osteoblastogenesis and mitigating bone loss, providing a feasible route for the development of therapeutic PTH analogs. Notably, compared with classic PTH(1-34), the synthetic monomeric peptide ^R25C^PTH(1-34) ^(^65) exhibits moderately reduced binding affinity for PTH1R and lower cAMP signal efficacy *in vitro*. Under chronic infusion in mice, ^R25C^PTH(1-34)induces only mild changes in serum calcium and phosphate, suggesting its potential to maintain desired receptor activation while minimizing calcium-phosphate-related adverse effects and widening the therapeutic safety window. From the perspective of tissue engineering and bone regeneration, incorporation of PTH-related peptides into 3D-printed porous titanium alloy scaffolds (66) has been shown to significantly enhance implant osteointegration even under osteoporotic conditions.

In conclusion, research on PTH and its associated molecular pathways, along with innovative therapeutic approaches, provide multidimensional advances for the intervention of bone metabolic diseases. Comprehensive mechanistic elucidation and targeted optimization have not only expanded the therapeutic potential of PTH in bone remodeling and OP management, but also laid a solid foundation for future development of safer and more effective bone regeneration strategies.

### 3.5 LGR receptor family: key nodes in the regulation of endocrine-metabolic-bone homeostasis

Among classical hormone membrane receptors (67, 68), the leucine-rich repeat-containing G protein-coupled receptor (LGR) family occupies a prominent position. LGR1 (follicle-stimulating hormone receptor), LGR2 (luteinizing hormone receptor), and LGR3 (TSH receptor) are essential for endocrine and metabolic regulation. As a newer member of this family, LGR4 has been identified as a critical player in multiple endocrine and metabolic disorders (69). LGR4 enhances Wnt/β-catenin signaling and is involved in the development and maintenance of the musculoskeletal system in both embryonic and adult stages (70). It orchestrates the differentiation and activity of osteoblasts and osteoclasts, with inactivating mutations such as p.R126X (71) being strongly linked to reduced BMD and an elevated risk of osteoporotic fractures in regions including the hip and spine. LGR4 also associates with bone metastasis in malignancies (69).

Mechanistically, LGR4 (67) can bind directly to human RANKL, competitively inhibiting the interaction between RANKL and RANK, thereby suppressing RANK-mediated osteoclast differentiation. Moreover, recent evidence suggests that the RSPO3–LGR4 axis (67) promotes osteogenic differentiation of human adipose-derived stem cells, while upregulation of LGR4 (69) may contribute to the attenuation of post-traumatic osteoarthritis progression. Collectively, LGR4 presents as a potential target for the treatment of bone metabolic disorders and for managing bone metastasis in cancer patients.

## 4 Axis of metabolic imbalance: interactions between glucose-lipid-uric acid homeostasis and bone fragility

Disruptions in glucose and lipid metabolism (72) are closely linked to increased bone fragility, significantly impacting fracture healing and the risk of subsequent fractures. It is important to emphasize that much of the current evidence linking metabolic imbalance to bone health primarily comes from preclinical models or observational epidemiological studies. These studies reveal correlations rather than universally applicable causal mechanisms. Therefore, caution should be exercised when extrapolating these findings to all patients with OP. Clinical studies have indicated that (73) type 2 diabetes mellitus adversely affects postoperative recovery following vertebral compression fractures, suggesting that metabolic complications independently and modifiably influence bone healing and refracture risk. Even in populations without diabetes (74), metabolic abnormalities are associated with compromised skeletal health. For example, metformin (75) has been shown to activate the Wnt/β-catenin pathway, offering a mechanistic basis for targeting metabolic pathways in the management and prevention of diabetes-associated OP. Moreover, studies have shown that (76) high-glucose (HG) mice can develop low-turnover OP, accompanied by a reduction in gut microbiota diversity. The gut microbiota of HG mice exhibits dysbiosis, particularly characterized by a decrease in *Bifidobacterium pseudolongum* and an increase in decanoic acid levels. Decanoic acid exacerbates the onset of OP by promoting Th17 differentiation and IL-17A production. Transplantation of fecal microbiota from normal mice into HG mice, supplementation with *Bifidobacterium pseudolongum*, or inhibition of IL-17A all mitigate bone loss, suggesting that supplementation with *Bifidobacterium pseudolongum* and inhibition of IL-17A are potential therapeutic strategies for high-glucose-induced OP.

Lipid metabolism also plays a central role in OP risk. Metabolic syndrome, characterized by central obesity and metabolic dysfunction, is recognized as an important risk factor for reduced bone mineral density (BMD) (77). In conditions such as metabolic syndrome, lipid metabolism disorders (78–81) (e.g., adipose tissue dysfunction, elevated free fatty acid levels, enhanced lipid peroxidation) exacerbate bone loss by inducing chronic inflammation, activating oxidative stress, and disrupting the energy sensing and signaling pathways between osteoblasts and osteoclasts. Numerous studies report complex relationships between various lipid markers and skeletal health (77), and these markers are additionally correlated with an increased fracture risk (82). Moreover, lipid accumulation indices (83) display a nonlinear inverse relationship with all-cause mortality among individuals with OP or reduced bone mass, supporting the potential utility of lipid phenotypes for prognostic stratification.

For phenotypic evaluation and risk identification, MRI-derived bone marrow proton density fat fraction (PDFF) (84) can distinguish individuals with vertebral fractures from those without, indicating PDFF as a potential imaging biomarker of bone fragility and providing a practical tool for the clinical assessment of the bone–adipose metabolic phenotype. Metabolomic studies have identified differential metabolic profiles (85, 86) among those with OP or low bone mass. The integration of lipidomics with imaging biomarkers may enable the development of comprehensive models for personalized risk assessment and therapeutic monitoring in the future.

Beyond glucose and lipid metabolism, the impact of uric acid (UA) on bone health has garnered increasing attention. Current evidence suggests that UA exhibits a dual role in OP (87): extracellular UA acts as an antioxidant by scavenging plasma free radicals (88), though its beneficial effect may be constrained by the lipid microenvironment of cell membranes. In contrast, intracellular UA, through its degradation, can generate free radicals and activate NADPH oxidase (89), intensifying oxidative stress, promoting inflammatory cytokine production, enhancing osteoclast-mediated bone resorption, and suppressing osteoblast-mediated bone formation. Additionally, UA (87) can inhibit vitamin D synthesis, leading to or exacerbating secondary hyperparathyroidism. Alterations in PTH may downregulate the urate transporter ABCG2, reducing UA excretion in the intestines and renal proximal tubules, thereby creating a detrimental metabolic feedback loop. Despite growing mechanistic understanding, findings from large cross-sectional studies (88–90) regarding the association of UA with OP and fracture risk remain inconsistent, highlighting the need for robust prospective studies and causal inference analyses.

## 5 The duet of bone homeostasis regulation: intercellular bridges of mitochondrial transfer and the metabolic hub role of the gut microbiota

In recent years, inter-organelle transfer, particularly mitochondrial transfer, has emerged as a prominent area of research. The pivotal role of mitochondria in cellular metabolism and differentiation has drawn significant attention to the mechanisms underlying mitochondrial transfer. Under pathological conditions (91), mitochondrial dysfunction leads to an imbalance in mitochondrial homeostasis, disrupting the osteoblast-osteoclast equilibrium and consequently impairing bone homeostasis. Notably, metabolic disturbances (92–95) (such as hyperglycemia and lipotoxicity) are important upstream factors contributing to mitochondrial dysfunction in bone cells. These metabolic abnormalities induce excessive production of mitochondrial reactive oxygen species (ROS) through mechanisms such as disruption of the electron transport chain and impairment of mitochondrial biogenesis. This disruption is a key pathogenic mechanism in OP, a chronic metabolic bone disorder. Musculoskeletal stromal cells (MSCs) (96) repair energy metabolism and restore function by transferring mitochondria to damaged or immune cells. Based on these findings, mitochondrial transfer has also opened new avenues for the treatment of inflammatory diseases and bone metabolic disorders.

Studies have shown that (97) macrophages can transfer mitochondria to mesenchymal stem cells (MSCs) in the bone marrow. When mitochondria derived from LPS-induced M1 pro-inflammatory macrophages are infused into mice, symptoms resembling OP are induced. However, infusion of mitochondria from normal sources effectively mitigates bone loss in OVX mice. This suggests that mitochondrial transfer may influence bone homeostasis and disease progression by regulating the interaction between macrophages and MSCs.

Furthermore, PINK1 (98), a key mitochondrial serine/threonine kinase, plays a critical role in mitochondrial quality control and autophagy. Downregulation of PINK1 significantly impairs osteoblast differentiation, induces mitochondrial dysfunction, increases reactive ROS, and disrupts calcium handling. Elevated ROS not only serve as direct mediators of oxidative damage but also act as important signaling molecules, capable of activating pro-inflammatory signaling pathways such as NF-κB (99) and the NLRP3 inflammasome (100, 101). This ‘oxidative-inflammation’ cycle, triggered by mitochondrial dysfunction, establishes a persistent pro-inflammatory state in the cellular microenvironment, thereby upregulating key factors such as RANKL and driving osteoclastogenesis, which skews the bone remodeling balance toward bone resorption. Experiments have shown that (98) PINK1 expression is notably reduced in the bones of OP patients. Mechanistically, PINK1 promotes osteoblast differentiation by regulating mitochondrial function and inhibiting ROS production. Meanwhile, the FoxO family (102) serves as a central transcription factor regulating cell death, proliferation, metabolism, and ROS clearance. FoxO3 can translocate to the nucleus, where it induces the transcription of antioxidant stress genes, such as Sod2 and Cat, thereby alleviating the abnormal accumulation of ROS.

The aforementioned studies have elucidated the critical role of mitochondrial transfer and related proteins in bone metabolism at the cellular and molecular levels. However, when the focus shifts from the cellular to the systemic level, it becomes evident that the gut microbiota and its metabolic products are also indispensable regulators of bone homeostasis. This suggests that the balance of bone metabolism is a process shaped by both microscopic cellular events and the broader systemic environment. Recent studies have shown that selenium-enriched egg white (103) can alleviate glucocorticoid-induced bone loss through gut microbiota-driven inhibition of Htr1b. Selenium-rich foods enhance antioxidant capacity and regulate multiple metabolic pathways, such as tryptophan metabolism, tyrosine metabolism, and arginine biosynthesis. Microbial tryptophan (104) can effectively improve metabolic diseases by modulating microbial communities. Its metabolites, indole-3-acetic acid (IAA) and indole-3-propionic acid (IPA), can repair the intestinal barrier integrity in OVX-induced osteoporotic mice and significantly mitigate bone loss in an aryl hydrocarbon receptor -dependent manner. Furthermore, supplementation with IAA and IPA enhances the function of M2 macrophages, promoting the secretion of the anti-inflammatory cytokine IL-10. This effect extends from the gut-associated lymphoid tissue (GALT) to the bone marrow, ultimately stimulating osteoblastogenesis and inhibiting osteoclastogenesis both *in vivo* and *in vitro*.

## 6 Conclusion

OP, a prevalent metabolic bone disorder, has historically been understood primarily through the lens of bone-mineral metabolic imbalance. However, accumulating evidence increasingly reveals that the onset and progression of OP are governed by a complex interplay among immune, endocrine, and metabolic axes. Synthesis of current research demonstrates that interactions across these axes provide novel perspectives and potential therapeutic targets for the prevention and treatment of OP. However, we must also recognize that the current understanding of the multi-axis interaction mechanisms still largely relies on animal models and observational association studies. Definitive clinical causal evidence and treatment outcomes for humans need to be further validated through large-scale prospective studies.

To facilitate the translation of this systemic understanding into clinical practice, we have tentatively proposed a classification framework for OP subtypes based on dominant pathophysiological axes (e.g., immune-driven, endocrine-driven, metabolic-driven, and mixed types). This framework aims to provide conceptual guidance for future mechanistic exploration, biomarker development, and the formulation of individualized therapeutic strategies.

However, the true application of the multi-axis regulatory theory in clinical settings faces several key challenges: First, how to integrate multi-omics biomarkers, such as those related to immunity, metabolism, and the gut microbiome, and validate their predictive value for fracture risk in large-scale prospective cohorts beyond traditional bone mineral density measures. Second, designing safe and effective multi-target combination therapies, which requires overcoming complex issues like drug interactions and individual heterogeneity. Finally, there is an urgent need to establish interdisciplinary clinical assessment and management pathways to systematically integrate multi-axis information and guide patient stratification and intervention.

Future investigations should focus more on elucidating the mechanisms underlying inter-axis communication and exploring their translational potential in clinical settings. As precision medicine and personalized therapy continue to advance, multidimensional strategies that integrate immune, endocrine, and metabolic interventions offer promising avenues for breakthroughs in OP management.
